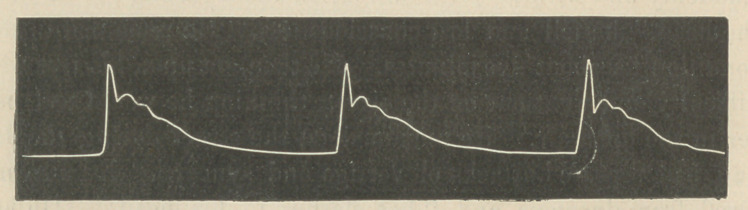# Cook County Hospital Service of T. Bidwell, M. D.

**Published:** 1884-01

**Authors:** C. E. Currie

**Affiliations:** Des Moines, Iowa


### Hospital Reports.

ARTICLE VIII.

COOK COUNTY HOSPITAL. SERVICE OF T. BIDWELL, M.D. MOTOR DERANGEMENT OF THE HEART—SYNCOPAL ATTACKS —DEATH—AUTOPSY. Reported by C. E. CURRIE, M.D., Des Moines, Iowa.

A. S.; German; male; occupation, cook; admitted to hospital Aug. 14, 1883.

Patient's mother, previous to her death, had two attacks similar to his present illness. Otherwise his family history is good. He uses alcoholic beverages moderately. No venereal history.

Patient had always enjoyed good health prior to August 1. He then had several attacks of syncope and dyspnœa, but gradually recovered. Had never noticed symptoms of trouble previous to that time. No dyspuœa; no palpitation of heart.

Present illness began at 5:00 A.M., on day of admission. Suddenly and with no premonition, except a feeling of "heat in the head," he fell and lost consciousness. He remained in this condition for about five minutes. He then got upon his feet with difficulty and went out on the street, thinking he would feel better in the open air. He remained on the street during the day and had repeated attacks of vertigo and syncope. He also suffered from dyspnœa and "hot flashes to his head." He had slight cephalalgia. In the evening he was picked up by a police patrol wagon and brought to the hospital.

On admission he complained only of "heat in the head" and dyspnœa. Pulse, eighteen per minute, full and strong. Half an hour afterwards, when he had been placed in bed, his pulse was twenty-eight. Temperature normal. Gave him atropiæ sulphas, gr. 1–60th, hypodermically, without producing any change in the pulse. The next morning he complained of slight dyspnœa and vertigo. Pulse, twenty-four.

On examination find patient strong and well-nourished. Skin pale and moist, especially about the head. Conjunctivæ slightly injected. Pupils normal. His neck is very short and thick.

*Thorax*—Respiration hurried and superficial. No adventitious sounds.

*Heart*—Apex beat in normal situation. It cannot be felt distinctly by palpitation. There is also a distinct pulsation in the third interspace to the right of the left nipple. Area of heart dullness not increased. Slight blowing murmur with systole heard over base of heart and transmitted a short distance into the carotid and subclavian arteries. The action of the heart is slow and labored. Twice, while the stethoscope was applied to the praecordial region, the heart ceased to beat for some seconds, and the patient placed his hand on his head and and said he " felt just as he did when he fainted."

*Abdomen*—Somewhat distended. Percussion note tympanitic. No cerebral symptoms have been observed. Temperature (when taken) has been found nearly always subnormal. Respiration about twenty per minute.

A sphygmogram was taken Sept. 29, from the radial artery. Pulse, twenty-three. The following is a chart of his pulse rate, taken morning and evening, during August and September.

During the time he was in the hospital, the following drugs were prescribed : Belladonna, digitalis, strychniæ, sulph., sanguinaria, nitre, nitro-glycerine. phosphorus, whiskey, ammoniæ carb. and potass. iodide.

He was in the same condition during the early part of Octo ber. In the last two weeks he had several attacks of syncope and finally died suddenly Oct. 30.

PULSE RATES.

| | Morning. | Evening. | | Morning. | Evening. | | Morning. | Evening. |
|---|---|---|---|---|---|---|---|---|
| August 15 | ......... | 30 | Sept. 1 | 17 | 15 | Sept. 18 | 26 | 20 |
| " 16 | 28 | 32 | " 2 | 20 | 22 | " 20 | 20 | 18 |
| " 17 | 32 | 28 | " 3 | 17 | 22 | " 21 | 22 | 23 |
| " 18 | 32 | 28 | " 4 | 18 | 24 | " 22 | 21 | ......... |
| " 19 | 32 | 36 | " 5 | 18 | 22 | " 23 | 20 | 23 |
| " 20 | 20 | 30 | " 6 | 18 | 22 | " 24 | 19 | 23 |
| " 21 | 24 | 11 | " 7 | 24 | 20 | " 25 | 23 | 22 |
| " 22 | 18 | 18 | " 8 | 16 | 18 | " 26 | 18 | ......... |
| " 23 | 28 | 22 | " 9 | 20 | 24 | " 27 | 22 | 22 |
| " 24 | 21 | 15 | " 10 | 19 | 20 | " 28 | 24 | 28 |
| " 25 | 16 | 16 | " 11 | 24 | 22 | " 29 | 23 | 22 |
| " 26 | 19 | 16 | " 12 | 20 | 22 | " 30 | 23 | ......... |
| " 27 | 13 | 17 | " 13 | 20 | 22 | | | |
| " 28 | 15 | 18 | " 14 | 17 | 24 | | | |
| " 29 | 16 | 12 | " 15 | 18 | 23 | | | |
| " 30 | 20 | 18 | " 16 | 18 | 22 | | | |
| " 31 | 16 | ......... | " 17 | 19 | 20 | | | |

The autopsy was made by Dr. W. T. Belfield. The viscera of the abdominal and thoracic cavities were apparently normal. No lesions of brain or medulla were found. The pneumogastric nerves were also apparently normal.

---

### A RARE CASE OF POISONING BY ATROPINE.

The patient, a woman 67 years of age, and very debilitated, suffered from an iritis of the left eye. She was treated for four days by instillation of a one per cent. solution of atropine into the conjunctival sac. On the second day she noticed after the treatment a dizziness in the head and dryness in the throat. On the third day the same symptoms appeared for a short time. On the fourth day, after leaving the clinic she became unconscious on the street, and fell to the pavement. In the hospital she was found to be delirious, the hands moving in all directions, pulse 190, temperature *elevated*, the pupils of both eyes dilated. Later on, the jactation of all the muscles was so considerable that a large board had to be fastened to her bedstead to prevent her falling out the bed. In the night the temperature was yet over 100. The symptoms entirely disappeared after three days.—*Memorabilien.*

## Figures and Tables

**Figure f1:**